# Calcium regulation of *Pseudomonas aeruginosa* metabolism

**DOI:** 10.1128/aem.02419-25

**Published:** 2026-06-15

**Authors:** Mackenzie Hull, Michelle King, Abigail Decker, Marianna A. Patrauchan

**Affiliations:** 1 Department of Microbiology and Molecular Genetics, Oklahoma State University 539937, Stillwater, Oklahoma, USA; Danmarks Tekniske Universitet, Kgs. Lyngby, Denmark

**Keywords:** shikimate pathway, nucleotide metabolism, iron acquisition, central carbon metabolism, glycerol metabolism, calcium

## Abstract

*Pseudomonas aeruginosa* (*Pa*), a ubiquitous opportunistic pathogen, is notorious for causing diverse life-threatening infections, including lethal lung infections in patients suffering from cystic fibrosis (CF). *Pa’s* ability to adapt to various environments is largely attributed to its metabolic flexibility and extensive regulatory networks. Understanding the mechanisms driving *Pa* adaptation during colonization of the airways of CF patients is of critical clinical importance. Previous studies by our group and others have shown that elevated Ca^2+^, a characteristic feature of CF airways, positively regulates virulence factor production in *Pa* and triggers its transition to a biofilm mode of growth. To generate further insights into Ca^2+^-dependent adaptations in this pathogen, we aimed to elucidate the regulatory impact of Ca^2+^ on its metabolism. To characterize Ca^2+^-induced metabolic alterations, a global untargeted gas chromatography-mass spectrometry-based metabolomics study was performed for both intracellular and extracellular fractions of mid-log and stationary-phase *Pa* cultures. The altered metabolites were mapped to specific pathways, and selected transcriptional changes and phenotypic outcomes were assayed. Our findings showed that Ca^2+^ regulates pathways involved in central carbon metabolism, nucleotide synthesis, the shikimate pathway, and glycogen metabolism in both mid-log- and stationary-phase cells. Furthermore, pathways involved in sequestering iron were induced by Ca^2+^ during both growth phases. Overall, these results indicate that exposure to elevated Ca^2+^, as within the CF airways, leads to metabolic adjustments and supports *Pa* survival in this environment.

**IMPORTANCE:**

Individuals with the genetic disorder, cystic fibrosis (CF), are predisposed to developing chronic, life-threatening *Pseudomonas aeruginosa* (*Pa*) infections. Elucidating the mechanisms that mediate the adaptation of *Pa* to the CF airways is essential for the development of new therapeutics to treat these infections. Previously, we have shown that elevated Ca^2+^ levels, such as those detected in CF airways, induce the production of multiple virulence factors, contributing to the overall pathogenicity of *Pa*. Here, we report that Ca^2+^ regulates *Pa* metabolism, affecting multiple pathways, including central carbon, nucleotide, and shikimate pathways. These metabolic alterations contribute to significant physiological outcomes, some of which are pertinent to *Pa* virulence and survival within the host. These findings suggest that Ca^2+^ can serve as a host factor that plays a significant role in *Pa* patho-adaptation to CF airways.

## INTRODUCTION

*Pseudomonas aeruginosa* (*Pa*), a ubiquitous opportunistic pathogen, possesses an outstanding ability to adapt to diverse environments, enabling the organism to colonize and cause a wide range of infections, including lung infections in cystic fibrosis (CF) patients, ventilator-associated pneumonia, urinary tract infections, burn wound infections, and keratitis (1, 2). Due to *Pa*’s resistance to host immune protection and antibiotics, its infections are becoming increasingly difficult to treat, and oftentimes impossible to eradicate (reviewed in references 3, 4). Such is the case of severe chronic CF lung infections, where colonization by *Pa* commonly worsens the prognosis, leading to progressive pulmonary decline and eventually death (5).

Understanding the molecular mechanisms of *Pa* adaptation to the host has been a long-standing challenge in the field. It is understood that upon invasion, *Pa* encounters and senses host factors and, in response, alters the expression of genes that aid in its survival (6). Many of these genes are involved in the production of virulence factors, such as exotoxins, lipopolysaccharide (LPS), proteases, pyocyanin, pyoverdine, and pili (reviewed in references 4, 6). Although a large body of research has been centered on virulence factor production and function, less attention has been focused on the adaptive metabolic rearrangements. Recent advances in metabolomic approaches have elucidated the intricate connectedness between virulence and metabolism in bacterial pathogens, demonstrating that alterations in one affect the other (7–10). Examples include metabolic rearrangements in *Klebsiella pneumoniae* and *Pa* in response to human serum and CF sputa, respectively, leading to increased production of siderophores in *K. pneumoniae* and quorum-sensing (QS) molecules in *Pa*, with the latter regulating the biosynthesis of different virulence factors (11–14).

The integration of metabolism and virulence is particularly relevant in the airways of CF patients, where the reduced efficiency of mucus clearance, elevated levels of carbohydrates, phospholipids, DNA, and amino acids (15, 16) promote the growth of invading pathogens (15). Supporting host-environment-guided metabolic adaptations, *Pa* CF clinical isolates show significantly altered metabolic profiles, including preferred catabolism of amino acids as carbon sources and increased accumulation of intracellular trehalose (11, 17–19). In addition to organic molecules, CF causes perturbations in ion homeostasis, leading to the accumulation of several ions, such as Ca^2+^, Fe^3+^, Mg^2+^, and Na^2+^ (20–22). Compared to healthy individuals, the levels of Ca^2+^ detected in CF nasal secretions, saliva, and sputum are elevated up to 5 mM (21–25), with cases of Ca^2+^ mineralization (26, 27), possibly reflecting the presence of even higher concentrations of Ca^2+^ in the airways. Whereas the significance of Fe in bacterial physiology has been recognized (28), the impact of Ca^2+^ remains understudied.

In eukaryotes, where the role of Ca^2+^ signaling and regulation is well established, it is known to control essential cellular processes, such as cell growth, differentiation, apoptosis, and immune responses (29–31). A growing body of research supports the significant role of Ca^2+^ in prokaryotes as well. Our group and others have shown that, although not essential for growth, Ca^2+^ has a significant impact on gene expression and physiology of *Pa* (32–35). We have shown that in response to elevated Ca^2+^ concentrations, *Pa* increases the production of multiple virulence factors, including pyoverdine, pyocyanin, and secreted proteases (33, 35). Additionally, Ca^2+^ triggers *Pa’s* switch to a biofilm mode of growth (33). Aiming to elucidate the underlying molecular mechanisms, we identified several key components of the Ca^2+^ signaling and regulatory network responsible for Ca^2+^-dependent gene expression in *Pa* (36, 37). Collectively, the findings suggest that Ca^2+^ may function as a host-derived signal that is sensed by *Pa*, resulting in adaptations via altered gene expression and adjusted physiology.

Despite the reported impact of Ca^2+^ on *Pa* gene expression and virulence, its role in metabolic regulation remains largely unexplored. To investigate the impact of elevated Ca^2+^ on *Pa* metabolism*,* we utilized a global untargeted metabolomics approach, a powerful tool in the -omics repertoire that, by identifying and quantifying metabolites, generates a direct snapshot of cellular activities. The intracellular and extracellular metabolites of mid-log- and stationary-phase cells were extracted, identified, and quantified by GC/MS. To delineate the physiological responses to Ca^2+^, we mapped the detected metabolites to specific metabolic pathways, followed by transcriptional and phenotypic assays to determine the biological implications of the affected pathways. We showed that in response to elevated Ca^2+^, *Pa* undergoes significant perturbations in several key metabolic pathways involved in central carbon metabolism, nucleotide biosynthesis, the shikimate pathway, and glycerol and glycogen metabolism during exponential and stationary growth phases. Overall, these results indicate that exposure to elevated Ca^2+^ modulates core metabolic routes in *Pa*, potentially facilitating its effective adaptation to Ca^2+^-rich host environments.

## RESULTS AND DISCUSSION

### Elevated Ca^2+^ significantly alters the *Pa* metabolome

**Fig 1:**
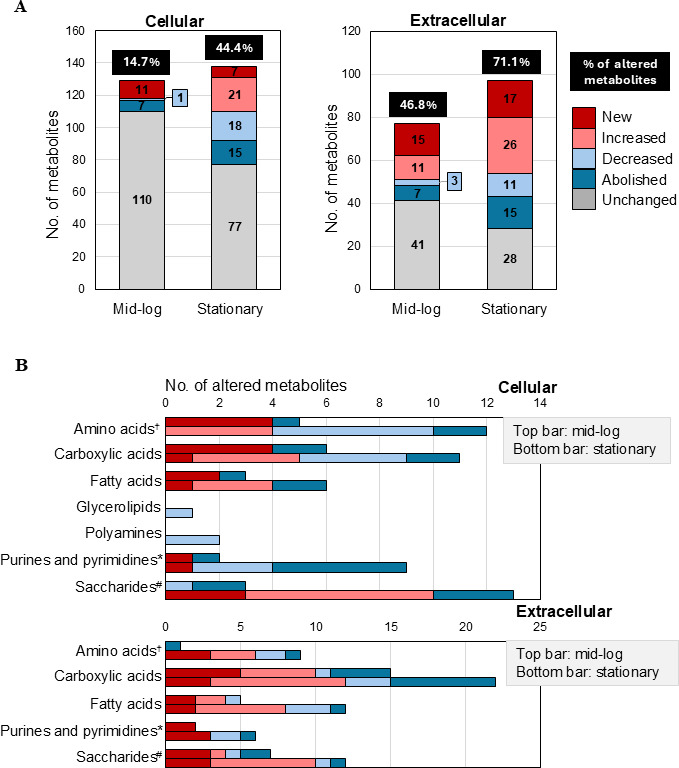
Global Ca^2+^-induced metabolic shifts in *Pa*. (**A**) Statistically significant (*P <* 0.05) Ca^2+^-affected cellular and extracellular metabolites in mid-log- and stationary-phase cells. Metabolites were detected, identified, and quantified by global non-targeted GC/MS and grouped based on Ca^2+^-dependent changes in abundance into new (present only at 10 mM Ca^2+^, dark red), increased (≥0.58 LFC, light red), decreased (≤−0.58 LFC, light blue), and abolished (absent at 10 mM Ca^2+^, dark blue). LFC refers to log_2_ fold-change. Percentages of statistically significantly altered metabolites were calculated based on the total number of detected metabolites in each growth condition and are depicted above the bars in black. Statistical significance was determined by a Welch’s t-test. The insignificantly altered metabolites were categorized as “unchanged”. (**B**) Chemical categories of significantly Ca^2+^-affected cellular and extracellular metabolites in mid-log- (top bar) and stationary-phase (bottom bar) cells. Metabolites were sorted into chemical categories using the NCBI PubChem database. The Ca^2+^-affected metabolites were assigned as new, increased, decreased, or abolished based on their response to Ca^2+^ as in A. Chemical categories in which no metabolites were detected are not shown. ^☨^ Includes modified amino acids, * includes nucleobases, nucleotides, and nucleosides, and ^#^ includes sugar alcohols.

To study the effect of Ca^2+^ on *Pa* metabolism, we used a global untargeted metabolomics approach based on gas chromatography-mass spectrometry (GC/MS) as reported in other metabolic studies in *Pa* (38–41). *Pa* PAO1 cells were grown in the absence or presence of 10 mM Ca^2+^ to mid-log and stationary phases, and their cellular and extracellular fractions were collected for follow-up extraction and analysis. This Ca^2+^ concentration was chosen to reflect elevated levels of the ion in CF airways and induce a robust metabolic response that would allow the changes to be accurately detected and mapped to specific pathways. A total of 186 metabolites were identified. The partial least squares discriminant analysis (PLS-DA) showed distinct separation of samples collected during mid-log and stationary phases and in the absence or presence of Ca^2+^, with each condition clustering consistently across replicates (see Fig. S1A at https://doi.org/10.5281/zenodo.20415982). According to the analysis, the abundances of 11 and 36 intracellular metabolites were significantly (*P* < 0.05) altered by Ca^2+^ during mid-log and stationary phase, respectively (Fig. 1A). The corresponding numbers of Ca^2+^-altered extracellular metabolites included 32 and 69 during mid-log and stationary phase, respectively (Fig. 1A). To understand the Ca^2+^-dependent metabolic alterations, the significantly altered metabolites were sorted based on changes in their abundance in response to Ca^2+^. Although untargeted metabolomic approaches are typically semi-quantitative in nature and obtaining quantitative accuracy is a common challenge (42, 43), we aimed to capture the biologically meaningful alterations of the metabolites by calculating ratios (fold change) between the averaged abundances at 10 mM Ca^2+^ vs 0 mM Ca^2+^ detected in four unpaired independent biological replicates. For quantitative comparisons outlined below, we have sorted the metabolites into four groups: “new” that were present only in the 10 mM Ca^2+^ condition, “increased” that met the threshold in log_2_ fold change (LFC) ≥ 0.58, “decreased” (≤−0.58 LFC), and “abolished” that were absent at 10 mM Ca^2+^. We considered a metabolic pathway to be impacted by Ca^2+^ if at least two associated metabolites were significantly altered. Ca^2+^ appears to promote a stronger regulatory impact on metabolism during the stationary phase, as the numbers of Ca^2+^-altered intracellular and extracellular metabolites in stationary-phase cells were 23.8% and 29.5% higher, respectively, than in mid-log phase cells.

All the differentially abundant (*P* < 0.05) cellular and extracellular metabolites (new, increased with LFC ≥0.58, decreased with LFC ≤−0.58, and abolished) were categorized based on their chemical structures (Fig. 1B). In agreement with the above observed trend, most chemical categories included a greater number of metabolites with altered abundances during the stationary phase (Fig. 1B); however, the distribution of positively and negatively regulated metabolites aligned more closely with their chemical category. For example, cellular purines and pyrimidines showed a large portion either decreased or abolished in the presence of Ca^2+^, specifically during the stationary phase (Fig. 1B). In contrast, the levels of cellular saccharides showed Ca^2+^-dependent increases during the stationary phase with only decreased abundances during mid-log phase (Fig. 1B). The observed differences in metabolic responses to Ca^2+^ in mid-log- vs stationary-phase cells may indicate a role for Ca^2+^ in fine-tuning the transition to the stationary phase. For instance, the increase in saccharides may reflect a shift from a more active metabolism to that of a slower one, which favors the storage of carbon, commonly associated with entry into the stationary phase (44, 45). Similarly, decreased levels of purines and pyrimidines are consistent with reduced proliferation or transcriptional activity during the stationary phase (46).

In addition to quantification, global metabolomics studies often face the challenge of distinguishing whether changes in metabolite abundances reflect their altered production or consumption. Therefore, to determine the biological significance of selected metabolite alterations, we aimed to delineate their physiological outcome(s), which are presented below. For this study, we maintained our main focus on understanding the intracellular metabolic alterations; therefore, most of the presented work below reflects the intracellular metabolic alterations.

### Ca^2+^ modulates glycerol metabolism and its incorporation into central carbon metabolism

**Fig 2:**
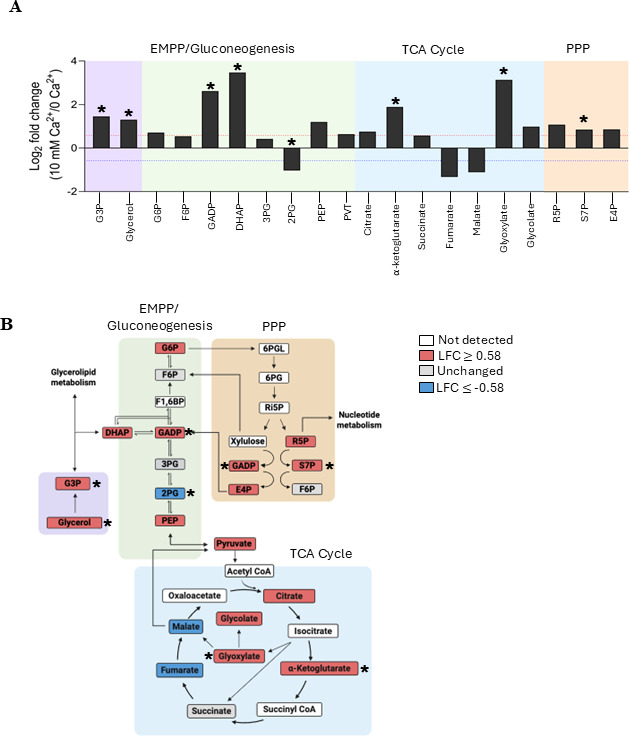
CCM is significantly altered by Ca^2+^ during stationary phase. (**A**) Ca^2+^-dependent changes in the abundances of CCM metabolites during stationary phase. LFCs were calculated for the ratios (10 mM/0 mM Ca^2+^) of the averaged abundances based on four unpaired independent biological replicates. The background colors correspond to the respective metabolic pathways depicted in B. ND, not detected. Red line, LFC = 0.58; blue line, LFC = −0.58. (**B**) Schematics depicting the effect of Ca^2+^ on CCM pathways during stationary phase. White, not detected; red, LFC ≥ 0.58; blue, LFC ≤ −0.58; gray, unchanged. Generated in Biorender.

Central carbon metabolism (CCM) consists of integrated biochemical pathways essential for cellular growth and function. In *Pa,* the canonical CCM backbone includes the Entner-Doudoroff pathway (EDP), the tricarboxylic acid (TCA) cycle with the glyoxylate shunt, and the pentose phosphate pathway (PPP), each producing distinct anabolic precursors (47). Pseudomonads, including *Pa*, possess an interrupted Embden-Meyerhof-Parnas pathway (EMPP) due to the absence of 6-phosphofructo-1-kinase (48, 49), preventing the phosphorylation of fructose-6-phosphate (F6P) to fructose-1,6-bisphosphate (F1,6BP). However, the lower half of the EMPP, starting from the dephosphorylation of F1,6BP to glyceraldehyde-3-phosphate (GADP) as well as gluconeogenesis, including the dephosphorylation of F1,6BP to F6P, remains functional. Glycerol, the structural backbone of phosphatidylcholine (a major component of airway surfactant), can be released during phosphatidylcholine degradation and serve as a significant carbon source for *Pa* during infection (50)*,* feeding into the CCM (48). Therefore, this study was designed with glycerol as the main source of carbon. In cellular fractions, 20 metabolites, including glycerol and glycerol-3-phosphate (G3P), were detected from the canonical CCM (Fig. 2A and B). All 20 CCM metabolites showed altered abundances (LFC ≥ 0.58, ≤-0.58) in response to elevated Ca^2+^ either at mid-log or stationary phase (Fig. 2A). Since the majority of them showed significant (*P* < 0.05) alterations during the stationary phase of growth (Fig. 2A) with less significant changes during mid-log (see Fig. S2A and B at https://doi.org/10.5281/zenodo.20415982), the impact of Ca^2+^ on stationary-phase metabolism will be discussed in greater detail below.

As depicted in Fig. 2B, glycerol is phosphorylated to glycerol-3-phosphate (G3P) and then oxidized to dihydroxyacetone-phosphate (DHAP), which can enter the EMPP or be used for the biosynthesis of glycerolipids (51). During the stationary phase, the Ca^2+^-dependent increase in G3P (1.45 LFC) (Fig. 2A and B) likely generated from uptaken glycerol, enters into the CCM through the conversion to DHAP, which is also increased in response to Ca^2+^ (3.47 LFC), along with other upper CCM metabolites: GADP (2.62 LFC) and G6P (0.71 LFC), and the PPP metabolites R5P (1.08 LFC), S7P (0.85 LFC), and erythrose-4-phosphate (0.86 LFC) (Fig. 2A and B). The elevated levels of R5P may reflect a reduced flow of this precursor into the nucleotide biosynthetic branch (discussed later). Furthermore, the levels of the TCA cycle intermediates, α-ketoglutarate (1.89 LFC) and glyoxylate (3.13 LFC), were statistically significantly increased in the presence of Ca^2+^ in stationary-phase cells. Although not statistically significant, the TCA cycle intermediates citrate (0.75 LFC) and glycolate (0.98 LFC) were also increased, while the abundance of both fumarate (−1.33 LFC) and malate (−1.11 LFC) was decreased (Fig. 2A and B) under these conditions. Together with the increased level of pyruvate (0.64 LFC), this suggests that 10 mM Ca^2+^ supports a potentially enhanced flow into the glyoxylate shunt in stationary-phase cells. This metabolic route is known to conserve carbon through bypassing CO_2_-generating steps and provide redox balance by limiting NADH production (52), which is particularly beneficial for mitigating oxidative stress and during the entry to the stationary phase, as illustrated in multiple bacterial species, including *Pa* (53–57). The Ca^2+^ induction of the glyoxylate shunt is in agreement with the previously reported Ca^2+^-dependent enhancement of *Pa* tolerance to oxidative stress (37). In addition, according to our reported RNA-seq analysis performed at 5 mM Ca^2+^ (accession: PRJNA874094, [35]), this level of Ca^2+^ induces several *Pa* genes associated with reactive oxygen species (ROS) detoxification, *sodM*, *katB*, and *ahpBCF* (58) (see Fig. S2C at https://doi.org/10.5281/zenodo.20415982). Taken together, these suggest that by activating the glyoxylate shunt and inducing ROS detoxification genes, Ca^2+^ helps *Pa* mitigate host-derived or self-produced oxidative stress, ultimately enhancing its survival within the host. This is especially pertinent within the CF lung, which is characterized by the increased infiltration of neutrophils generating oxidative bursts to destroy pathogens (reviewed in [59]). In agreement, the upregulation of the glyoxylate shunt genes has been reported in CF isolates (50, 60).

**Fig 3:**
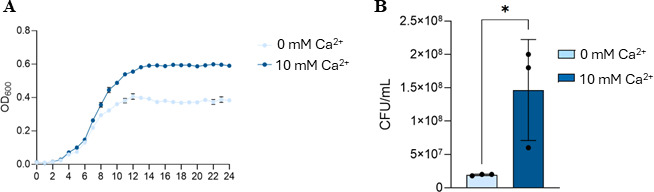
Ca^2+^ enhances growth level in *Pa.* (**A**) Growth of *Pa* in BMM at 0 mM Ca^2+^ or 10 mM Ca^2+^ monitored by OD_600_ for 24 h at 37°C in a SynergyMx BioTek plate reader. Pre-cultures were inoculated with *Pa* grown on LB from frozen stocks and incubated at 37°C until mid-log phase (12 h). Following normalization to OD_600_ 0.3, they were used to inoculate 1:1,000 main cultures in BMM containing 0 mM Ca^2+^ or 10 mM Ca^2+^. Error bars indicate the standard deviation of three independent biological replicates. (**B**) CFU of *Pa* grown to mid-log (12 h) as in A. Culture aliquots were serially diluted to 10^−8^ in 0.85% NaCl (saline), and 50 µL of each dilution was plated on LB plates and incubated at 37°C overnight to calculate CFUs. Error bars indicate the standard deviation of three independent biological replicates. Statistical significance was determined using an unpaired Student’s t-test. * indicates *P* < 0.05.

Considering that CCM is essential for growth, the observed Ca^2+^-dependent increase in several CCM metabolites may reflect the activation of catabolic pathways leading to an elevated growth level. Supporting this hypothesis, we observed that the presence of Ca^2+^ increased cell density of *Pa* cultures measured by OD_600_ by 22% and 51% at mid-log (8 h) and early stationary (14 h) phases, respectively (Fig. 3A). Since, according to TEM micrographs, the cell size was not affected by growth in the presence of Ca^2+^ (see Fig. S3A at https://doi.org/10.5281/zenodo.20415982), we concluded that the Ca^2+^-dependent increase in OD_600_ was not due to differences in cell size. To elaborate, we also quantified the number of cells in mid-log cultures grown in 0 mM and 10 mM Ca^2+^ conditions by colony-forming units (CFUs). In agreement with the growth data, the number of cells in the presence of 10 mM Ca^2+^ was 9.8-fold greater than at 0 mM Ca^2+^ (Fig. 3B). It is possible that the presence of Ca^2+^ promotes the stochastic rise of glycerol-metabolizing cells discovered in *P. putida* KT2440 (61) and shifts a sub-population of glycerol-dependent metabolic persisters toward metabolic activity, particularly during the transition to the stationary phase, thereby enhancing the overall glycerol utilization and increasing cell densities.

Overall, these data suggest that Ca^2+^ regulates the fine-tuning of the CCM, particularly in cells transitioning to the stationary phase, which favors gluconeogenesis and the glyoxylate shunt. The activation of the glyoxylate shunt during the stationary phase, as well as during infection in the CF airways, has been reported (54, 57, 62, 63).

### The shikimate pathway and downstream aromatic amino acid and secondary metabolite biosynthetic pathways are impacted by Ca^2+^ in a growth phase-dependent manner

**Fig 4:**
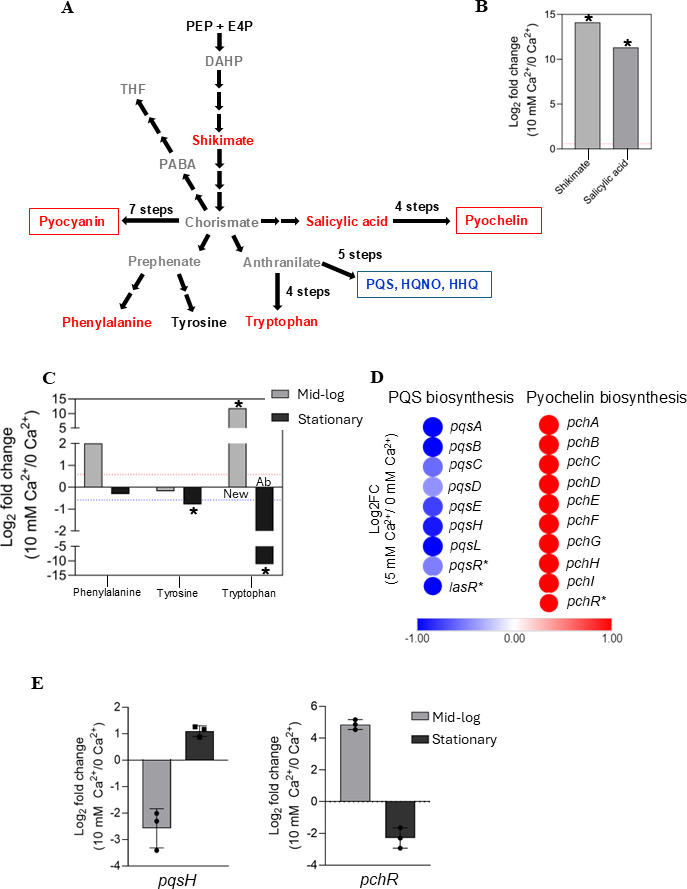
The shikimate pathway and downstream aromatic amino acid and secondary metabolites biosynthetic pathways are impacted by Ca^2+^ at mid-log. (**A**) Shikimate metabolic pathway and downstream secondary metabolites, aromatic amino acid biosynthetic pathways, and their metabolites and products are shown as Ca^2+^-increased (red) and Ca^2+^-decreased (blue) at mid-log phase. Undetected metabolites are indicated in gray. Unchanged metabolites are indicated in black. The secondary metabolites depicted in boxes indicate the impact of Ca^2+^ that was assessed based on extraction assay (33) and RNA seq transcriptional analysis of the corresponding biosynthetic genes (accession: PRJNA874094 [35]). (**B**) Ca^2+^-dependent changes in cellular shikimate and salicylic acid abundances at mid-log phase. Shikimate and salicylic acid were only detected in 10 mM Ca^2+^ in mid-log phase. LFCs were calculated for the ratios (10 mM/0 mM Ca^2+^) of the averaged (*n* = 4 independent biological replicates) abundances. The red dashed horizontal line indicates 0.58 LFC. Metabolites that were not detected are indicated as ND. (**C**) Ca^2+^-dependent changes in cellular aromatic amino acid abundances at mid-log and stationary phases (*n* = 4 independent biological replicates). Tryptophan was only detected in the 10 mM Ca^2+^ condition at mid-log phase (depicted as “NEW”) and only in the 0 mM Ca^2+^ condition at stationary phase (depicted as “AB,” abolished). To plot these data as LFC, a value of 0.01 (below the lowest detected abundance of 0.4) was assigned to tryptophan in the conditions where it was not detected. The red and blue dashed horizontal lines indicate 0.58 and −0.58 LFC, respectively. * indicates *P <* 0.05 as determined by Welch’s t-test. (**D**) Ca^2+^-dependent changes (5 mM Ca^2+^/0 mM Ca^2+^) in expression of PQS and pyochelin biosynthesis genes at mid-log phase as determined by RNA-seq (*n* = 3 independent biological replicates) (accession: PRJNA874094 [35]). Asterisk (*) indicates positive transcriptional regulators. (**E**) Ca^2+^-dependent changes (10 mM Ca^2+^/0 mM Ca^2+^) in the expression of *pchR* and *pqsH* during mid-log and stationary phases, as determined by RT-qPCR (*n* = 3 independent biological replicates).

The shikimate pathway is a core metabolic pathway that produces chorismate, the intermediate required for biosynthesis of aromatic amino acids as well as several key aromatic virulence factors (reviewed in reference 64). The pathway (Fig. 4A) begins with the conversion of erythrose-4-phosphate (E4P, produced in the PPP) and phosphoenolpyruvate (PEP, produced in the EMPP) to form 3-deoxy-D-arabinoheptulosonate 7-phosphate (DAHP) followed by six steps leading to the production of chorismate (64). Once formed in *Pa,* chorismate can be used to produce: (i) prephenate, the precursor for phenylalanine and tyrosine; (ii) anthranilate, the precursor for tryptophan and the QS molecules PQS, HQNO, and HHQ (65); (iii) siderophore pyochelin; (iv) pyocyanin; or (v) tetrahydrofolate, an essential cofactor. From the four metabolites produced in the shikimate pathway, we detected shikimate, which was present only in the presence of Ca^2+^ at the mid-log phase (Fig. 4A and B). Additionally, salicylic acid, a derivative of the shikimate pathway and a precursor for pyochelin biosynthesis, was also detected only in the presence of Ca^2+^ at the mid-log phase (Fig. 4A and B). Further supporting a strong induction of the pathway by Ca^2+^ in mid-log cells, the abundance of tryptophan was significantly elevated. Also increased was phenylalanine, albeit not statistically significantly, with tyrosine remaining unchanged by Ca^2+^. On the other hand, tryptophan and tyrosine were significantly decreased by Ca^2+^ during the stationary phase of growth (Fig. 4A and C).

Biosynthesis of the 2-alkyl-4-quinolone QS molecules, PQS, HQNO, and HHQ, is carried out by enzymes encoded by the *pqsABCDE* operon, regulated by PqsR/MvfR (65, 66). As a part of the pathway, the oxidase PqsL produces HQNO (67), shown to have auto-poisoning and antibacterial effects (68, 69). HHQ is synthesized by PqsBC, which is further oxidized to produce PQS by PqsH (65). Although no QS molecules were detected in the metabolomic data, according to our RNA-seq transcriptional studies (accession: PRJNA874094 [35]), the expression of *pqsABCDE, pqsL, pqsH*, and *pqsR* was downregulated in mid-log cells in response to 5 mM Ca^2+^ (Fig. 4D). Furthermore, the expression of *lasR*, the transcriptional activator of the Las QS system that positively regulates *pqsABCDE* (70), was also downregulated by Ca^2+^ (Fig. 4D). To strengthen the evidence and determine the impact of 10 mM Ca^2+^ on the regulation of PQS biosynthesis genes, we applied reverse transcription-quantitative PCR (RT-qPCR) and measured the expression of *pqsH*, responsible for the last step in PQS biosynthesis, in mid-log cells. Supporting the previous data collected at 5 mM Ca^2+^, the expression of *pqsH* was decreased (−2.5 LFC) in the presence of 10 mM Ca^2+^ (Fig. 4E; also see Fig. S4A at https://doi.org/10.5281/zenodo.20415982). Interestingly, this Ca^2+^-dependent decrease in *pqsH* expression coincided with the stark Ca^2+^-dependent increase in cellular tryptophan abundance in the mid-log phase (Fig. 4C; also see Fig. S4B at https://doi.org/10.5281/zenodo.20415982). Taken together, these observations may reflect the re-direction of the shikimate pathway toward synthesizing aromatic amino acids and away from synthesizing PQS molecules. Considering that QS is most active at high cell densities, we also measured the expression of *pqsH* in the stationary-phase cells, where we observed a reduction during the transition from mid-log to stationary phase, but significantly less so in the presence of Ca^2+^ (Fig. S4A at https://doi.org/10.5281/zenodo.20415982), resulting in a higher level of *pqsH* expression (1.1 LFC) than that at no Ca^2+^ (Fig. 4E; also see Fig. S4A at https://doi.org/10.5281/zenodo.20415982). This higher level of *pqsH* expression in the presence of Ca^2+^ coincided with the decrease in tryptophan abundance during the stationary phase (Fig. 4C; also see Fig. S4B at https://doi.org/10.5281/zenodo.20415982), indicating possible reverse routing of the shikimate pathway back to biosynthesis of PQS. This growth-phase-dependent shift in Ca^2+^ regulation of the PQS-tryptophan biosynthetic branches, combined with the observed Ca^2+^ induction of the shikimate pathway, underscores the broader downstream impact of Ca^2+^ on PQS-controlled processes, including biofilm formation (71, 72), iron uptake (73, 74), outer membrane vesicle (OMV) formation (75), pyocyanin production (76), as well as interactions of *Pa* with the host and other microorganisms (77–79).

**Fig 5:**
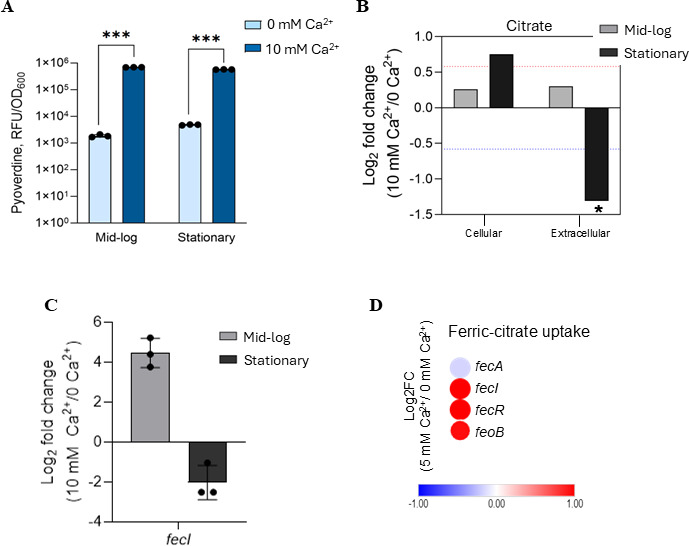
Ca^2+^ stimulates iron uptake mechanisms. (**A**) Pyoverdine production was quantified by measuring fluorescence (excitation 400 nm, emission 460 nm) in mid-log (12 h) and stationary (24 h) phase *Pa* cultures grown in BMM containing 0 mM Ca^2+^ or 10 mM Ca^2+^ (*n* = 3 independent biological replicates). The relative fluorescent units (RFU) were normalized by optical density (OD_600_) of the corresponding cultures. Statistical significance was evaluated by Student’s t-test, *** indicates *P* < 0.0001. (**B**) Ca^2+^-dependent changes (10 mM Ca^2+^/0 mM Ca^2+^) in cellular and extracellular citrate abundances in mid-log and stationary-phase cells. LFCs were calculated for the ratios (10 mM/0 mM Ca^2+^) of the averaged (*n* = 4 independent biological replicates) abundances. The red and blue dashed horizontal lines indicate 0.58 and −0.58 LFC, respectively. * indicated *P-*value of < 0.05 as determined using a Welch’s t-test. (**C**) Ca^2+^-dependent changes (10 mM Ca^2+^/0 mM Ca^2+^) in *fecI* expression during mid-log and stationary phases as determined by RT-qPCR (*n* = 3 independent biological replicates). (**D**) Ca^2+^-dependent changes (5 mM Ca^2+^/0 mM Ca^2+^) in the expression of ferric-citrate uptake regulatory genes, *fecAIR*, and Fe^2+^ transporter gene, *feoB*, as determined by RNA-seq analysis (accession: PRJNA874094 [35]).

The biosynthetic pathway for pyochelin, the low-affinity iron-sequestering siderophore (80), begins with the conversion of chorismate to isochorismate by the salicylate biosynthesis isochorismate synthase, PchA, and then to salicylic acid via isochorismate pyruvate lyase, PchB. Salicylic acid is then shuttled into a four-step process to produce pyochelin (81) (Fig. 4A). We detected salicylic acid only in mid-log cells grown at elevated Ca^2+^ (Fig. 4A and B), indicating a strong induction by Ca^2+^. Further supporting Ca^2+^ induction of pyochelin biosynthesis, our RNA-seq data (accession: PRJNA874094 [35]) showed that the pyochelin biosynthesis genes, *pchABCD* and *pchEFGHI*, were significantly upregulated by 5 mM Ca^2+^, with the expression of their positive regulator, *pchR*, increased by 6.0 LFC (Fig. 4D). To elaborate on this induction by Ca^2+^, we determined the impact of 10 mM Ca^2+^ on pyochelin biosynthesis gene expression by using RT-qPCR and detected a 4.8 LFC increase in *pchR* expression compared to that at 0 mM Ca^2+^ during the mid-log phase (Fig. 4E). However, in the stationary phase, we observed a 2.3 LFC decrease in *pchR* expression in response to 10 mM Ca^2+^ (Fig. 4E), suggesting that in the presence of Ca^2+^, the cellular iron requirements may be more efficiently met during the exponential growth, thereby reducing the demand for pyochelin during stationary phase. A similar pattern was detected for *fecI* (Fig. 5C), the regulator of ferric citrate uptake, further supporting this interpretation. Consistent with these transcriptional changes, stationary-phase cultures of *Pa* showed a loss of salicylic acid, a direct precursor of pyochelin (82). Together, these findings highlight a growth phase-dependent role of Ca^2+^ in modulating pyochelin biosynthesis in *Pa*. Similarly, reduced expression of pyochelin biosynthesis genes during the stationary phase has also been reported during growth in artificial sputum medium (83). Additionally, fewer sputum samples from CF patients contained pyochelin compared to pyoverdine, highlighting the switch in the production of these siderophores (84). This switch is likely implemented to ensure adequate iron acquisition by prioritizing the production of the higher-affinity siderophore, pyoverdine, during later stages of growth that coincide with iron-depleted conditions of mature infections (85).

Pyocyanin, another metabolic fate of chorismate, is produced through a seven-step process (Fig. 4A), involving the enzymes encoded by two phenazine biosynthetic clusters, *phzA1B1C1D1E1F1G1* and *phzA2B2C2D2E2F2G2,* along with *phzM* and *phzS* (86). Although no pyocyanin-related metabolites were detected in this study, our previous reports showed that 10 mM Ca^2+^ induced pyocyanin production in mid-log- and stationary-phase planktonic, as well as swarming *Pa* (33, 87). This highlights another branch of the shikimate pathway that is regulated by Ca^2+^, which leads to the production of a potent virulence factor, pyocyanin, responsible for a pleiotropic impact on *Pa* physiology (88, 89) and pathogenesis (90, 91), especially during chronic infection (92).

Overall, both metabolomic and transcriptional data indicate that the shikimate pathway, a universal metabolic hub essential for the production of aromatic amino acids and highly influential secondary metabolites, is regulated by Ca^2+^. The outcomes of Ca^2+^ regulation coincide with those observed during the transition from acute to chronic infection *in vivo*, indicating that Ca^2+^ is likely involved in potentiating this transition.

### Ca^2+^ stimulates iron uptake mechanisms in *Pa*

We have previously reported that the presence of 5 mM Ca^2+^ induces the expression of genes encoding for the biosynthesis of both siderophores in *Pa*, the low-affinity pyochelin and the high-affinity pyoverdine (35). Here, we show that exposing *Pa* cultures to 10 mM Ca^2+^ also stimulated the production of both pyochelin (discussed above as a part of the shikimate pathway (Fig. 4A, D and E)) and pyoverdine. The latter was measured by fluorescence during mid-log and stationary phases and showed an 8.5- and 6.9- LFC increase, respectively (Fig. 5A). This regulation is particularly important for *Pa* during infection, where free iron is not readily available as it is withdrawn by the host as a part of nutritional immunity (93). To obtain iron during CF infections, *Pa* increases the production of the two siderophores (predominantly, pyoverdine), which enhances the pathogen’s virulence (83, 84, 94, 95). Our data suggest that by inducing the production of these siderophores, elevated Ca^2+^ plays an important role as a host-derived signal to cue for the upregulation of siderophore production to enhance survival within the host.

In addition to siderophores, citrate, a key intermediate in the TCA cycle, has been increasingly recognized for its role in sequestering iron in both gram-positive and gram-negative bacteria, including *Pa* (96–101). As established in *E. coli*, once bound to Fe^3+^, the Fe^3+^-citrate complex is translocated across the outer and inner membranes by FecABCDE, whose expression is regulated by the sigma/anti-sigma factors, FecIR (102). *Pa* possesses a similar system, FecAIR, with FecIR reported to be induced by iron-limiting conditions, and the expression of the Fe^3+^-citrate receptor, FecA, is dependent on the presence of exogenous citrate (103). It has been proposed that the inner membrane Fe^2+^ transporter, FeoB, facilitates the import of Fe^2+^ released from the reduced Fe^3+^-citrate complex in the periplasm (100, 101). Our metabolomics data detected extracellular citrate, which was significantly decreased in the presence of Ca^2+^ by 57.0%, whereas the abundance of cellular citrate increased by 23.6% in stationary-phase *Pa* in response to 10 mM Ca^2+^ (Fig. 5B). This Ca^2+^-dependent perturbation was not observed in the mid-log phase cells, where the levels of cellular and extracellular citrate remained unchanged (Fig. 5B), further highlighting the significant impact of Ca^2+^ on *Pa* metabolism during the transition to stationary phase. The coinciding decrease in extracellular levels and increase in cellular levels of citrate may reflect the stimulation of Fe^3+^-citrate uptake. To assess the role of Ca^2+^ in regulating this mechanism, we quantified the expression of the anti-sigma factor, *fecI*, at different Ca^2+^ levels by using RT-qPCR. Contrary to our expectations, *fecI* expression was upregulated by Ca^2+^ (4.6 LFC) in mid-log cells and decreased (−1.6 LFC) in stationary-phase cells (Fig. 5C). The induction of *fecI* by 10 mM Ca^2+^ is consistent with the RNA-seq data showing a significant induction of *fecIR* genes in response to 5 mM Ca^2^ during mid-log (Fig. 5D). The decreased expression of *fecI* in the stationary phase by Ca^2+^ may reflect a diminished iron requirement that was likely satisfied during the exponential phase through activation of multiple iron-sequestering systems (described above). The unchanged expression of *fecA* in response to Ca^2+^ was not surprising, considering that its expression is dependent on exogenous citrate (103), which is not present in the medium. Furthermore, according to our RNA-seq data (accession: PRJNA874094 [35]) (Fig. 5D), the expression of the Fe^2+^ transporter, FeoB, was induced by Ca^2+^ (0.95 LFC), which indicates a possible involvement of this transporter in translocating Fe^2+^ from the reduced Fe^3+^-citrate complex. The observed differences in cellular and extracellular citrate abundance during the stationary phase may be due to the cooperative activity of not-yet-characterized Fe^3+^-citrate transporters and FeoB, warranting further studies.

### Nucleotide metabolism is altered by elevated Ca^2+^

**Fig 6:**
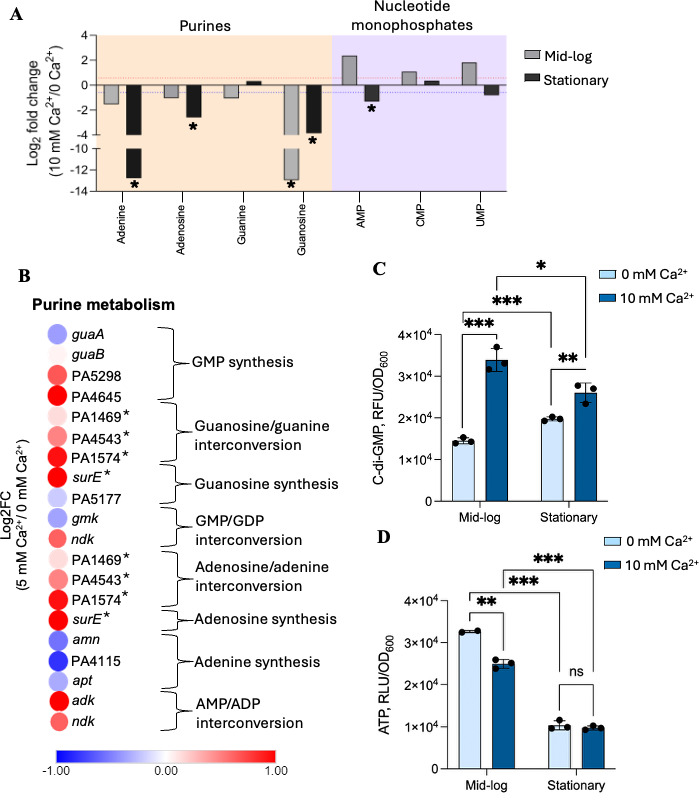
Nucleotide metabolism is altered by Ca^2+^. (**A**) Ca^2+^-dependent changes (LFC of 10 mM Ca^2+^/0 mM Ca^2+^) in cellular abundances of purines (orange) and nucleotide monophosphates (purple) at mid-log and stationary phases. Adenine and guanosine were only detected at 0 mM Ca^2+^ (depicted as “AB”, abolished) during stationary and mid-log phases, accordingly. To report these data as LFC, a value of 0.01 (below the lowest detected abundance of 0.4) was assigned to adenine and guanosine in conditions where they were not detected. * indicated *P-*value of < 0.05 as determined using a Welch’s t-test. (**B**) Ca^2+^-dependent changes (LFC of 5 mM Ca^2+^/0 mM Ca^2+^) in the expression of key purine metabolic genes at mid-log phase as determined by RNA-seq analysis (accession: PRJNA874094 [35]). The purine metabolism genes were categorized using the KEGG database and grouped by function. * indicates genes involved in both guanosine/guanine and adenosine/adenine metabolism. (**C**) Intracellular cyclic-di-GMP levels were quantified using the pCdrA::*gfp* reporter by measuring fluorescence (excitation 485 nm, emission 535 nm) in mid-log (12 h) and stationary (24 h) phase *Pa* cells grown in BMM containing with or without 10 mM Ca^2+^ (*n* = 3 independent biological replicates). The RFUs were normalized by optical density (OD_600_) of the corresponding cultures. Statistical significance was evaluated by Student’s t-test, ** indicates *P* < 0.001, *** indicates < 0.0001. (**D**) Intracellular ATP levels were quantified using the BacTiter-GLO Microbial Cell Viability Assay in mid-log (12 h) and stationary (24 h) phase *Pa* cells grown in BMM with or without 10 mM Ca^2+^ (*n* = 3 independent biological replicates). The RFUs were normalized by optical density (OD_600_) of the corresponding cultures. Statistical significance was evaluated by Student’s t-test, ** indicates *P* < 0.001.

Nucleotides are the foundational building blocks of DNA and RNA . In addition, the purines, adenosine and guanosine, are the key components of high-energy carriers, ATP and GTP, as well as signaling molecules, including cAMP and cyclic-di-GMP (c-di-GMP). Due to their essential roles in cellular life, the biosynthesis of nucleotides and their end products is tightly controlled, and their dysregulation is highly consequential (reviewed in reference 80). Additionally, nucleotide biosynthesis is essential for survival and production of virulence factors in human pathogens, including *Pa* (7, 9, 104–108). Our metabolomics analysis revealed that in the presence of 10 mM Ca^2+^, the abundance of the purine guanosine was significantly decreased in both the mid-log (−13.3 LFC) and stationary phase (−2.7 LFC) cells. In addition, adenosine (−2.7 LFC) and adenine (−13.0 LFC) were also significantly decreased or abolished in response to Ca^2+^ during stationary phase (Fig. 6A). In contrast, although not statistically significant, the pyrimidines, uracil and thymine, along with the precursor, uridine, remained unaffected by Ca^2+^ during mid-log with smaller changes during the stationary phase (see Fig. S5A at https://doi.org/10.5281/zenodo.20415982). The abundance of cytosine was decreased in the presence of Ca^2+^ during the mid-log (−1.57 LFC) but not the stationary phase (Fig. S5A at https://doi.org/10.5281/zenodo.20415982). Interestingly, the abundances of the DNA and RNA building blocks, adenosine-monophosphate (AMP) (*P* < 0.05), cytidine-monophosphate (CMP), and uridine-monophosphate (UMP), were elevated in the mid-log in response to Ca^2+^ (Fig. 6A), which may reflect the observed Ca^2+^-enhanced growth (Fig. 3A). According to our RNA-seq data (accession: PRJNA874094 [35]), the expression of the genes involved in adenosine and guanosine was induced by 5 mM Ca^2+^ in mid-log cells (Fig. 6B). Furthermore, a partial Ca^2+^ induction of the genes involved in the production of purine precursor 5-aminoimidazole-4-carboxamide ribonucleotide (FAICAR) was also observed (see Fig. S5B at https://doi.org/10.5281/zenodo.20415982). On the other hand, the genes responsible for adenosine and guanosine incorporation into DNA synthesis were downregulated in response to Ca^2+^ (Fig. S5B at https://doi.org/10.5281/zenodo.20415982). Taken together, the Ca^2+^ transcriptional induction of purine biosynthesis during mid-log and the reduction of purine levels during both phases may reflect several cellular circumstances, including a possible lack of the purine precursors (amino acids glutamine, glycine, aspartate, and folate derivatives), ATP-intensive purine *de novo* synthesis (reviewed in reference 109), and high purine turnover for signaling (via (p)ppGpp, cAMP, and c-di-GMP) and DNA repair (reviewed in reference 110). Among these, we tested the levels of the signaling molecule, c-di-GMP, and the high-energy carrier, ATP, at different Ca^2+^ levels. As expected, the level of c-di-GMP assessed using the pCdrA::*gfp*(ASV)^C^ reporter (111) increased with higher cell density during the stationary phase at no added Ca^2+^ condition (Fig. 6C). In the presence of 10 mM Ca^2+^, c-di-GMP levels increased 2.3-fold at mid-log (12 h) compared to that at no Ca^2+^. The difference declined slightly during the stationary phase but remained at least 30% higher in the presence of Ca^2+^ (Fig. 6C). This Ca^2+^-dependent increase in c-di-GMP may reduce the cellular pool of available guanosine, the precursor for c-di-GMP. On the other hand, this signaling molecule is a potent regulator of the transition from a planktonic to a biofilm mode of growth (reviewed in reference 112). The regulatory role of Ca^2+^ in this transition has also been established (33, 34). Increased biofilm formation is a hallmark characteristic of chronic *Pa* lung infections (reviewed in reference 113), further highlighting the role of Ca^2+^ in potentiating the patho-adaptation of *Pa.* In addition to stimulating biofilm formation, c-di-GMP has been reported to positively regulate swarming motility and pyocyanin production (33, 114). To determine whether Ca^2+^ impacts the regulation of (p)ppGpp production, we assessed the expression levels of *spoT* and *relA* in response to Ca^2+^, which are required for the molecule’s regulation and biosynthesis (115). The corresponding LFCs were −0.5 and −0.6 for *spoT* and *relA*, respectively, suggesting that the observed diminished abundances of guanosine are not due to the production of (p)ppGpp.

We also measured the intracellular ATP levels using the BacTiter-GLO Microbial Cell Viability Assay (Promega). The ATP levels significantly decreased in mid-log cells at 10 mM Ca^2+^ and further decreased in the stationary-phase cells grown at both Ca^2+^ levels (Fig. 6D). The Ca^2+^-dependent reduction of ATP levels in mid-log cells may reflect the elevated energy usage supporting the Ca^2+^-enhanced growth (Fig. 3A). The reduced levels of ATP may not be sufficient to support the ATP-intensive *de novo* synthesis of purines leading to their decreased abundance (Fig. 6A). Further studies will address the impact of Ca^2+^ on the levels of (p)ppGpp and cAMP and examine how this modulation influences the metabolic reprogramming and stress adaptation in *Pa.*

### Ca^2+^ stimulates trehalose production in stationary-phase cells

**Fig 7:**
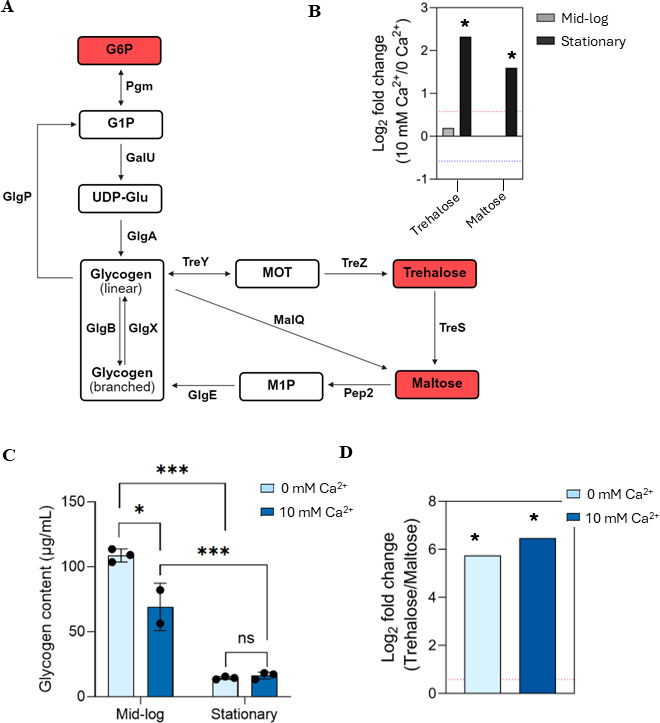
Ca^2+^ regulates glycogen metabolism. (**A**) Glycogen biosynthesis pathway and its metabolites. The increase in abundances of both trehalose and maltose (>0.58 LFC) in 10 mM Ca^2+^ at stationary phase is indicated in red. (**B**) Ca^2+^-dependent changes (LFC of 10 mM Ca^2+^/0 mM Ca^2+^) in cellular trehalose and maltose abundances during mid-log and stationary phases at 0 mM and 10 mM Ca^2+^. Maltose was not detected (ND) at either Ca^2+^ condition in mid-log phase. * indicated *P-*value of < 0.05 as determined using a Welch’s t-test. (**C**) Ca^2+^-dependent changes in glycogen production. Glycogen was extracted from mid-log and stationary *Pa* cells grown at 0 mM and 10 mM Ca^2+^ and quantified using the glycogen-iodine reaction assay (*n* = 3 independent biological replicates). Statistical significance was evaluated by Student’s t-test, * indicates < 0.05, ns indicates not significant. (**D**) The isomerization of trehalose to maltose was evaluated by generating a ratio of trehalose (Tre) to maltose (Mal) abundances at 0 mM and 10 mM Ca^2+^ in stationary-phase cells. * indicated *P-*value of < 0.05 as determined using a Welch’s t-test.

Trehalose is a disaccharide consisting of two glucose monomers linked with an α-1,1-glycosidic bond. In *Pa*, trehalose production is involved in the ethanol and osmotic stress responses, where it serves as a compatible solute, protecting the membrane and cellular proteins from degradation (116–118). In addition, trehalose is directly involved in the metabolism of glycogen, used to store carbon (116, 119–122), and can facilitate bacterial infection (123). In some bacteria, trehalose is synthesized *de novo* from G6P via the OtsAB pathway (124–126); however, this pathway is not present in Pseudomonads (127). In *Pa*, trehalose can be produced through the degradation of glycogen by TreYZ and then isomerized by TreS to maltose, which can also be produced directly from glycogen by MalQ or used for its synthesis by Pep2 and GlgE (116) (Fig. 7A). Glycogen can also be metabolized by GlgP to glucose-1-phosphate (G1P) (Fig. 7A), which, however, was not detected in our data, and therefore is not addressed here. Our metabolomic analysis revealed that the abundances of trehalose (2.32 LFC) as well as maltose (1.60 LFC) were significantly increased at elevated Ca^2+^ in stationary-phase cells but remained unchanged or not detected during mid-log (Fig. 7B). To determine whether the accumulation of trehalose was a by-product of glycogen degradation, we quantified cellular levels of glycogen using the glycogen-iodine reaction method (128) and a standard curve generated based on purified glycogen (Invitrogen) (see Fig. S6 at https://doi.org/10.5281/zenodo.20415982). In mid-log cells, glycogen levels decreased 33.1% in response to Ca^2+^; however, in stationary-phase cells, although overall significantly declined, they were not affected by Ca^2+^ (Fig. 7C). We also calculated the ratios of trehalose and maltose averaged abundances in stationary-phase cells at each Ca^2+^ concentration (Fig. 7D), which showed an increase from 5.75 LFC at no Ca^2+^ to 6.47 LFC at 10 mM Ca^2+^. Together, these findings indicate that Ca^2+^ stimulates the production of trehalose and maltose during the stationary phase, possibly through activating catabolism of glycogen and reducing the isomerase activity of TreS. Importantly, it has been reported that *Pa* CF isolates can accumulate exorbitant amounts of trehalose and transition to utilizing trehalose instead of glycine-betaine as an osmolyte, which coincides with the transition to mucoid phenotypes, predominant during infection (17, 117). The production of glycogen in CF clinical isolates has been attributed to the TreSYZ pathway and related to their multidrug resistance (116, 129). Of further importance, trehalose has been shown to scavenge ROS, such as H_2_O_2_ (130), which may provide an additional mechanistic route for the previously reported role of Ca^2+^ in enhancing the survival of *Pa* under oxidative stress (37).

**Fig 8:**
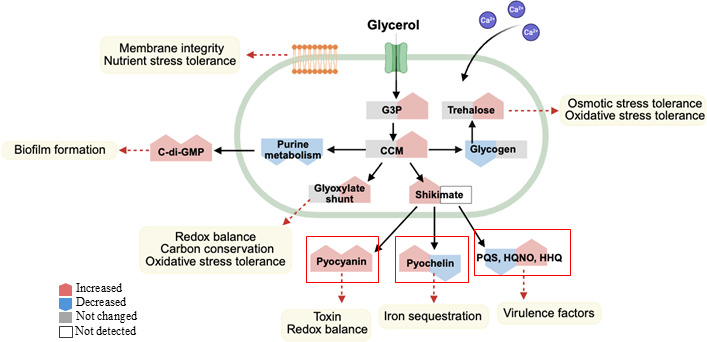
The summary of Ca^2+^ impact on *Pa* metabolism during mid-log and stationary phases. Altered metabolic pathways are shown with some of their key metabolites and products. The impact of Ca^2+^ is indicated by red arrows (increased), blue arrows (decreased), and gray boxes (unchanged) at mid-log (left) and stationary phase (right). Undetected metabolites in the shikimate pathway at stationary phase are indicated by a white box. Red outlines indicate that the Ca^2+^ impact on gene expression for pyocyanin, pyochelin, and PQS/HQNO/HHQ biosynthesis was assessed based on transcriptional analysis, either by RT-qPCR or RNA-seq analysis (accession: PRJNA874094 [35]). The positive impact of Ca^2+^ on the production of pyocyanin has been previously reported (33). The physiological outcomes of the pathways are indicated with red dashed arrows. According to the metabolomic data, during mid-log phase, Ca^2+^ activated shikimate and purine metabolism. Transcriptional and functional studies verified increased c-di-GMP, pyocyanin, and siderophore production, and decreased production of PQS, HQNO, HHQ, and glycogen by Ca^2+^. During stationary phase, Ca^2+^ activated CCM (including the glyoxylate shunt) and further enhanced purine metabolism. The levels of trehalose and c-di-GMP were increased by Ca^2+^. These alterations may promote biofilm production and increase tolerance to osmotic and oxidative stresses, collectively enhancing the overall virulence and persistence of *Pa* in the CF airways.

During infection, *Pa* is continuously inundated with a multitude of environmental stressors, including oxidative and osmotic stress (118, 131, 132). The coexistence of these conditions with elevation of Ca^2+^ during CF may present a selection pressure supporting *Pa* adaptive mechanisms leading to increased growth and resistance in the presence of Ca^2+^. As summarized in Fig. 8, our data show that Ca^2+^ affects multiple metabolic pathways in *Pa*, including central carbon, nucleotide, and shikimate, some of which contribute to the production of virulence factors and survival within the host. These findings suggest that Ca^2+^ serves as a host-derived signal that induces preemptive responses to environmental stressors in *Pa*, promoting its patho-adaptation.

## MATERIALS AND METHODS

### Bacterial strains, media, and growth conditions

*Pa* PAO1 was grown in biofilm minimal media (BMM) (33), which consists of 50 mM glycerol, 9.0 mM sodium glutamate, 0.15 mM NaH_2_PO_4_, 0.34 mM K_2_HPO_4_, and 145 mM NaCl. The medium was adjusted to pH 7.0 and autoclaved. Following sterilization, trace metals, vitamins, and MgSO_4_ were added to achieve a final concentration of 4 µM CuSO_4_, 3.48 µM ZnSO_4_, 3.6 µM FeSO_4_, 5.06 MnCl_2_, 4.1 nM biotin, 1.88 µM thiamine, and 0.02 mM MgSO_4_. When needed, the medium was supplemented with 5 mM or 10 mM CaCl_2_•2H_2_O. When higher density cultures were required, BMM8 containing 0.08 mM MgSO_4_ was used instead of BMM. Frozen stocks were streaked on LB agar and incubated overnight at 37°C. Individual colonies were used to inoculate pre-cultures that were grown in BMM (no Ca^2+^) for 12 h and then normalized to OD_600_ 0.3. Normalized cultures were inoculated 1:1,000 into 100 mL of BMM containing no or 10 mM Ca^2+^ and grown at 37°C and 200 rpm for either 12 h or 24 h, to reach the mid-log or stationary phase, respectively. The timing of the growth phases was determined by monitoring OD_600._

### Metabolomics sample preparation

PAO1 cultures (four unpaired independent biological replicates) were grown in 100 mL of BMM (in 250-mL flasks) containing no or 10 mM Ca^2+^ for either 12 h (mid-log phase) or 24 h (stationary phase) (see Fig S1B at https://doi.org/10.5281/zenodo.20415982). Cultures were harvested by centrifugation at 10,000 × *g*, 4°C, for 5 min, and both the intracellular and extracellular fractions were collected. The supernatants were filter-sterilized to remove any remaining cells. The cell pellets were washed once with phosphate-buffered saline (PBS), spun again, then resuspended in 500 µL PBS and combined with 500 µL of 2:1 (vol/vol) chloroform: methanol. The samples were aliquoted in 1 mL volumes and shipped to the Roy J. Carver Biotechnology Metabolomics Center for analysis.

### GC/MS analysis

Pellets were extracted with 1 mL of 70% MeOH by ultrasound treatment using Qsonica Q700 sonicator. Lysates were centrifuged for 10 min at 18,000 rpm at 4°C, and supernatants were transferred to new tubes and evaporated under vacuum. To achieve the primary goal of metabolic alterations, a single internal standard (hentriacontanoic acid, 1 mg/mL) was used for normalization. The internal standard (30 µL) was added to each sample prior to derivatization and then derivatized with 100 µL of methoxyamine hydrochloride (40 mg/mL in pyridine) for 90 min at 50°C, then with 100 µL MSTFA at 50°C for 120 min. Samples were analyzed at the Roy J. Carver Biotechnology Metabolomics Center using a GC/MS system (Agilent Inc, Palo Alto, CA, USA) consisting of an Agilent 7890 gas chromatograph, an Agilent 5975 mass selective detector, and a HP 7683B autosampler. Gas chromatography was performed using a ZB-5MS (60 m × 0.32 mm I.D. and 0.25 μm film thickness) capillary column (Phenomenex, CA, USA). The mass spectrometer was operated in positive electron impact mode (EI) at 69.9 eV ionization energy in m/z 30–800 scan range. Artificial peaks were identified by incorporating “method blanks” consisting of LC/MS-grade water (100 µL) spiked with the internal standard, which underwent identical processing as experimental samples (evaporation under vacuum and derivatization). Peaks detected in method blanks were subtracted from all samples to remove background contamination. Additionally, all “column bleeds” peaks generated by the column’s stationary phase (5% phenylmethylpolysiloxane) were also subtracted from the data prior to analysis. MS peaks were evaluated by AMDIS 2.71 (NIST, Gaithersburg, MD, USA) program, and metabolites were identified by a custom-built library (484 unique metabolites). To allow comparison between samples, all data were normalized to the internal standard in each chromatogram and the sample dry weight.

### Metabolomics analysis

Metabolite abundances were reported as dry weight per 1 mL internal standard. All analyses were performed using the Metaboanalyst 6.0 platform (https://www.metaboanalyst.ca/), following similar methods described in reference 133. Briefly, a partial least squares discriminant analysis (PLSDA) was performed to distinguish the differences between the 0 mM and 10 mM Ca^2+^ conditions in both the intracellular and extracellular fractions. To determine the effect of Ca^2+^ on metabolite regulation, a multivariate analysis was performed using the log_10_-transformed normalized values. When metabolites were not detected in a specific condition, to calculate fold changes, they were given a placeholder value of 0.01 (below the lowest detected abundance of 0.04). The mean metabolite abundances from four unpaired independent biological replicates at 10 mM Ca^2+^ were divided by the mean metabolite abundances at 0 mM Ca^2+^ to generate a fold-change. Metabolites were considered altered by Ca^2+^ if their fold changes (FC) were greater than 1.5, and, accordingly, log_2_ FC (LFC) were ≥0.58 and ≤−0.58 for increased and decreased, respectively. Metabolites were classified as “new” if they appeared only in the 10 mM Ca^2+^ condition or “abolished” if they were absent at 10 mM Ca^2+^. A Welch’s t-test was used to determine the statistical significance of the Ca^2+^-induced alterations. Metabolites were mapped to metabolic pathways using KEGG and BioCyc databases. Chemical structures were assigned to metabolites using the PubChem database.

### Quantification of colony-forming units

PAO1 cultures were grown in BMM with and without 10 mM Ca^2+^ until mid-log (12 h) phase. Cultures were serially diluted to 10^−8^ in 0.85% NaCl (saline), and 50 µL of each dilution was plated on LB plates and incubated overnight at 37°C. Colony-forming units were calculated as CFU/mL of culture.

### C-di-GMP quantification

Production of c-di-GMP was monitored using the c-di-GMP-inducible *cdrA* promoter construct (pCdrA::gfp(ASV)) expressing green fluorescent protein (GFP) (111). For this, overnight cultures of *Pa* were grown in BMM (without Ca^2+^) for 12 h, normalized to OD_600_ 0.3, and inoculated into fresh BMM with or without 10 mM Ca^2+^ at a 1:100 ratio. Diluted cultures were added to a black 96-well plate (Corning) and monitored for growth and GFP fluorescence (excitation 485 nm/emission 535 nm) for 24 h using the SynergyMx BioTek plate reader. Fluorescence was normalized by OD_600_. Experiments were performed in triplicate and repeated twice independently.

### ATP quantification

Pre-cultures of *Pa* were inoculated in BMM (without Ca^2+^), grown at 37°C, 200 rpm shaking for 12 h, then normalized to OD_600_ 0.3 and inoculated at a 1:1,000 ratio into BMM with or without 10 mM Ca^2+^. PAO1 cultures were grown to mid-log (12 h) or stationary (24 h) phase. Cultures were collected and normalized to the lowest OD_600_ (0.4–0.8 for mid-log and 1.2–1.6 for stationary-phase samples) in 1.5 mL of BMM. Normalized samples were diluted 1:10 and mixed with BacTiter-Glo Reagent (Promega) in a 1:1 ratio to achieve a total volume of 200 µL, which was added to a white 96-well plate (Greiner Bio-One). Upon mixing, treated cells were incubated at room temperature for 5 min, and then luminescence was measured using the SynergyMx BioTek plate reader. RLUs were normalized by OD_600_. An unpaired Student’s t-test was used to determine statistical significance.

### Glycogen quantification

Pre-cultures of *Pa* were inoculated in BMM8 (without Ca^2+^), grown at 37°C, 200 rpm shaking for 12 h, normalized to OD_600_ 0.3, and inoculated at a 1:1,000 ratio in BMM8 with or without 10 mM Ca^2+^. Cultures were grown to mid-log (12 h) or stationary (24 h) phase, pelleted at 9,190 × *g* for 10 min, and then pellets were resuspended in saline. Samples were transferred to 1.5 mL tubes, heated at 95°C for 10 min, and then sonicated for 20 s on/20 s off, five times. The samples were pelleted at 6,200 × *g* for 10 min, and 100 µL of the supernatants was added to wells of a clear 96-well plate (Greiner Bio-One). Absorbance was measured at 492 nm prior to adding 12 µL Lugol solution (Sigma-Aldrich) and after 1 min of incubation at room temperature with Lugol solution (128). The absorbances with no Lugol solution were subtracted from the absorbances with Lugol solution and then normalized by OD_600_. Glycogen concentrations were determined based on a standard curve generated by using commercially available purified glycogen (Invitrogen) at concentrations ranging from 0 to 150 µg/mL. The means were calculated from three biological replicates. An unpaired Student’s t-test was used to determine statistical significance.

### Pyoverdine quantification

Pre-cultures of *Pa* were inoculated in BMM (without Ca^2+^), grown at 37°C, 200 rpm shaking for 12 h, normalized to OD_600_ 0.3, and 200 µL of normalized culture was added per well of a black clear-bottom 96-well plate (Corning). Cultures were incubated at 37°C, 200 rpm shaking for 24 h. Fluorescence (400 nm/460 nm excitation/emission) (134) and OD_600_ were measured hourly. To quantify the amounts of pyoverdine produced, RFUs were normalized by OD_600_. Every experiment included three independent biological replicates and was repeated twice for consistency. An unpaired Student’s t-test was used to determine statistical significance.

### RNA isolation

PAO1 cultures were grown in BMM with or without 10 mM Ca^2+^ until mid-log (12 h, OD_600_ 0.18–0.21) or stationary (24 h, OD_600_ 0.36–0.46) phase. Immediately upon collection, cultures were mixed with RNA-later at a 1:1 ratio and harvested by centrifugation. Total RNA was extracted from cells using the RNeasy Mini Kit (Qiagen) and then treated with Turbo DNase (Invitrogen). The residual DNA was assessed using PCR with primers specific for the 16S rRNA gene. RNA was quantified using a NanoDrop spectrophotometer (Thermo Scientific), and the quality was assessed using both gel electrophoresis and UV absorption at 260 nm.

### Real-time quantitative PCR analyses

cDNA was synthesized from purified RNA using the Transcriptor First Strand cDNA Kit (Roche) and assessed by PCR using primers specific for the 16S rRNA gene (Table 1). Real-time PCR amplifications were carried out in a LightCycler 480 II (Roche) using primers specific for the housekeeping gene, *nadB* (PA0761), and experimental genes (Table 1). The relative expression of each targeted gene was quantified using the standard curve method (135) and normalized to that of *nadB*. The impact of Ca^2+^ on gene expression was reported as log2 fold of the ratio of normalized target gene expression at 10 mM Ca^2+^ to that at 0 mM Ca^2+^. An unpaired Student’s t-test was used to assess statistical significance. Experiments were executed using cDNA synthesized from RNA collected from three independent biological replicates.

**TABLE 1:** Strains, plasmids, and oligonucleotides used in this study

Strain/plasmid/oligonucleotide	Description	Reference
Strains		
PAO1	Laboratory wild-type strain of *P. aeruginosa*	(136)
PAO1/pCdrA::*gfp*(ASV)^C^	PAO1 with pCdrA::*gfp*(ASV)^C^	(111)
Plasmids		
pCdrA::*gfp*(ASV)^C^	c-di-GMP fluorescent reporter plasmid	(111)
Oligonucleotides		
16S_533_F	GTGCCAGCAGCCGCGGTAA	(137)
16S_1100_R	AGGGTTGCGCTCGTTG	(137)
pchR_525_F	CAGCGCACAGTTCCTTTCCG	This study
pchR_676_R	GTCAGCTTGCGCGGGTTCAT	This study
pqsH_159_F	CAGCACCCTGGATCTCGACC	This study
pqsH_349_R	GAATGCGCGACTCATCCAGC	This study
fecI_135_F	CGTCAAGGTCCTGGTTTCGC	This study
fecI_307_R	GGGTCTCTTCGCTGGGGAC	This study

### Transmission electron microscopy

Pre-cultures were inoculated into BMM with *Pa* grown on LB from frozen stocks and incubated at 37°C until mid-log phase (12 h). Cultures were normalized to OD_600_ 0.3 in BMM 0 mM Ca^2+^ and inoculated 1:1,000 in 25 mL of BMM containing 0 mM, 5 mM, or 10 mM Ca^2+^ in 125 mL flasks. Cultures were grown to mid-log phase (12 h) at 37°C and collected via centrifugation at 6,000 × *g* for 10 min. Pellets were resuspended and fixed in a 10× volume of 2% glutaraldehyde for two h at room temperature. Cell pellets were washed, resuspended in a 10× volume of cacodylate buffer, and incubated at room temperature for 15 min. Washes were repeated three times. Cell pellets were resuspended in 1% OsO_4_ and incubated at room temperature for 1 h. Cell pellets were washed in cacodylate buffer and incubated at room temperature for 15 min. Washes were repeated three times. Cell pellets were dehydrated in a series of ethanol washes followed by three washes using propylene oxide. Cell suspensions were incubated in propylene oxide overnight at room temperature. Cells were embedded in 100% embedding medium and incubated at 60°C and allowed to polymerize for 48 h. Micrographs were captured on the EOL JEM-2100 Scanning TEM (STEM) with Bruker Quantax 200 energy-dispersive X-ray microanalysis (EDS) system. TEM micrographs were used to quantify the cell size of *Pa* grown in BMM containing 0 mM Ca^2+^ or supplemented with 5 mM or 10 mM Ca^2+^. Measurements for length and width were recorded for at least four cells per condition.
